# Exploring the bounds of consumer choice in supported housing: A reflexive thematic analysis of data generated from supportive housing tenants in British Columbia, Canada

**DOI:** 10.1371/journal.pmen.0000505

**Published:** 2025-12-15

**Authors:** Tracy Smith-Carrier, Hannah Dahlquist-Axe, Denise Prindiville, Carrie Anne Marshall

**Affiliations:** 1 School of Humanitarian Studies, Royal Roads University, Victoria, British Columbia, Canada; 2 School of Humanitarian Studies, Royal Roads University, Victoria, British Columbia, Canada; 3 School of Humanitarian Studies, Royal Roads University, Victoria, British Columbia, Canada; 4 School of Occupational Therapy, Faculty of Health Sciences, Western University, London, Ontario, Canada; University of Western Australia, AUSTRALIA

## Abstract

Consumer choice is a key principle in Housing First and supported housing models. Through the provision of permanent housing and individualized supports, these models promote an empowerment and recovery-oriented approach that advances individual self-determination and overall wellbeing. The purpose of this study is to explore housing tenants’ experiences, with, and perspectives of, complex care and supportive housing models in British Columbia (BC), Canada to understand how well these models align conceptually with the notion of consumer choice. Viewed through a critical theoretical framework and applying a reflexive thematic analysis to interview data generated from housing tenants in BC, we constructed several themes that spoke directly or indirectly to dimensions of consumer choice. The essence of ‘constrained choice’ in these data suggest that the underlying components of consumer choice may be infrequently practiced in complex care and supportive housing programs in BC. Given the importance of consumer choice in improving the quality of life and wellbeing of supported housing tenants, work is necessary to broaden its scope and exercise in these contexts.

## Introduction

As housing becomes increasingly more unaffordable, the waitlists for housing support in the province of British Columbia (BC), Canada continue to grow [1]. BC Housing, responsible for fulfilling the directives of the BC Ministry of Housing and Municipal Affairs, aims to address homelessness and manage social and supportive housing programs in the province. The *Belonging in B.C. Homelessness Plan* details the provincial government’s plan to work “with Indigenous partners, people with lived experience of homelessness, local governments, service providers and other partners to create a province where everyone has a community and a place to call home” [2]. Housing First (HF) principles feature prominently in the homelessness plan, which emphasizes the need for immediate responses to homelessness, and the implementation of the Integrated Support Framework that seeks to coordinate and streamline the delivery of health and social supports to people at risk of losing their housing or who are unhoused. The framework advances a “new integrated and coordinated service model to deliver a suite of wraparound supports to improve stability, *choice/personal agency* and inclusion for people at risk of or experiencing homelessness across unsheltered and housing settings” [[2] (p16); emphasis ours]. This study explores housing tenants’ experiences with and perspectives of supportive and complex care housing models in BC and how well these models align with the notion of consumer choice.

Government housing programs in BC, Canada’s third most populated province, feature a variety of models, including but not limited to supportive housing and complex care housing. The *supportive housing* model offers “subsidized housing with on-site supports for single adults, seniors and people with disabilities at risk of or experiencing homelessness” [3]. Administered through non-profit housing providers, tenants are offered self-contained units and access to communal (and in some cases, culturally appropriate) spaces. Staff are available on-site 24/7 to provide a range of supports (e.g., life skills training, connections to health care, mental health or substance use services) to “help people find and maintain housing” [3].

*Complex care housing*, a relatively new offering in BC that is predominately a partnership between health authorities for clinical support and BC Housing for non-clinical support, seeks to support “people living with significant mental health, addictions, or concurrent challenges, and other functional needs, who are at risk of homelessness” [4]. Depending on the community and an individual’s preferences, complex care housing is offered in a range of formats, provided by non-profit housing organizations, including in small communal residences, supportive housing sites, or in market rentals with rent supplements. Irrespective of accommodation type, residents are provided with a comprehensive suite of “person-centred services to meet their needs”, inclusive of primary care, mental health and addictions services, social and cultural supports, peer programming and supports, and assistance with activities of daily living [4]. For this study, we focused on supportive housing and complex care housing as both offer subsidized rental fees and on- and/or off-site services. The study is timely given the dearth of literature on supportive housing models in BC, with even less research exploring their fit with the notion of consumer choice.

In the HF literature broadly and in research on supported/supportive housing models specifically, consumer choice is a core element that supports housing stability and consumer wellbeing. In contrast to research that locates the problem and solutions of ‘wicked problems’, such as homelessness, within the individual, as typically featured in bio-scientific and behavioural modification approaches [5], this research explores the current systems, institutions, policies, practices, and social relations that impact people dwelling in supportive housing units/buildings.

### Supported housing and HF models

The HF model was developed in the early 1990s, built on the principles of psychiatric rehabilitation and the core belief that housing is a basic human right [6]. HF, both a philosophy and programmatic approach [7], recognizes the importance of providing permanent housing with individualized supports to people experiencing homelessness without expectations that they meet any *a priori* conditions of housing readiness [8,9]. HF is a recovery-oriented approach that advances individual self-determination and overall wellbeing [10]. Before the introduction of HF, the prevailing Treatment First (TF) approach in housing delivery models typically required clients to either meet certain housing selection requirements, or to graduate through a series of steps, including expectations related to sobriety, drug consumption and/or mental illness status in order to be deemed housing ready [11]. Contrary to a responsive rights-based approach which poses on political actors “immediate obligations to address urgent violations” [12 [p6)] of the right to housing, TF approaches stymy entries into housing, and retention in it, by exacerbating clients’ mental health and/or prompting substance use relapses [13].

The underlying HF philosophy is that people are better able to work towards their personal goals and achieve a better quality of life if their basic human right to stable housing is met. HF principles include: (a) immediate access to permanent housing with no housing readiness conditions; (b) *self-determination and consumer choice*; (c) recovery orientation; (d) client-driven, individual supports; and (e) social and community integration [14; emphasis ours]. In HF, consumer choice reflects the notion that the client is an active participant in the choice of housing (e.g., type, location, etc.), course of treatment (before, after, or not at all), development of personal goals (e.g., education, training, wellness, etc.), and resources needed to achieve them [8].

In the context of supported/supportive housing specifically, there are a few housing models currently being employed, albeit with conflicting conceptual labels, definitions, programmatic elements, and measurements indices [15]. In juxtaposition to earlier models reflecting a custodial approach to housing individuals with mental illness (focused on maintenance), a supported housing approach has been applied more recently (focused on recovery). The latter offers tenants affordable housing integrated in the community, with flexible and individualized supports, to live independently [16].

The supported housing model emerged in the late 1980s, with the growing recognition that mental health consumers preferred not to live with other mental health consumers but rather independently, with flexible outreach support, not live-in support staff [15], as is characteristic of the custodial model. Wong et al. [17] outline the principles of the supported housing model as inclusive of: a home in the community as a basic right; normal roles as regular tenants and community members; consumer empowerment; and functional separation between housing and support services. Piat et al. [18] further contextualize these principles, including the signing of a lease guaranteeing full tenancy rights; lack of activity restrictions; rent limited to 30% of income; optional support services decoupled from housing eligibility; and the option to add services without risk to housing tenure. What Wong et al. [17] identify as one of the operational domains guiding supported housing principles, and Piat et al. [18] argue is central to recovery models, is the concept of consumer choice.

Piat et al. [18], summarizing Parkinson et al.’s [[19] (p306)] research, note that the cornerstones of supported housing relate to “choice, autonomy and normality,” the latter eschewing arrangements that could invoke stigma. Within this model, consumer choice reflects the centrality of personal values and preferences in the housing location (e.g., neighbourhoods, proximity to amenities, etc.), type (e.g., congregate or single occupancy), and structure (e.g., alone or with others). It is demonstrated by the availability of housing options open to the tenant and extent to which tenancy agreements reflect those found in the private rental market sector [17]. In a qualitative research study with 24 supported housing tenants with serious mental illness, Jost et al. [20] found that choice (e.g., to choose one’s own case manager) was a significant contributor to wellbeing and mental health recovery.

Supported housing models espousing a HF orientation do not require that tenants seek or receive mental health or substance use treatment to obtain or maintain housing [16]. Many now consider the supported housing model as an evidence-informed program under a more expansive HF paradigm, which includes a variety of housing arrangements within an integrated systems model of service delivery [21]. Both in HF and in the broader literature exploring the nuances of supported housing models, consumer choice appears to be a key and salient feature.

In the neoliberal era of ‘permanent austerity’ and limited resources [22], however, systemic constraints and pressures limit guarantees of choice in housing arrangements and individually tailored resources [8,23]. Additional barriers include public and political sentiments in relation to NIMBYism (not-in-my-backyard) including demands that housing sites for individuals experiencing homelessness be moved outside NIMBY-supporters’ neighbourhoods, discrimination by landlords (who may be less inclined to rent properties to people experiencing poverty and homelessness), land-use planning and zoning issues, and a chronic lack of affordable housing supply. In Canada, the responsibility for housing is shared across all levels of government. The provincial/territorial governments (and the local/municipal governments they contract out to) have sought to administer housing through a variety of programs, albeit with significant funding shortfalls [24]. Indeed, in the absence of an affordable housing market, consumer choice is invariably constrained [25].

### Theoretical framework

Critical theory represents a burgeoning umbrella of theoretical strands (e.g., critical disability studies, Queer theories, critical feminist, critical race, intersectionality, etc.) that is interdisciplinary, emancipatory, and transformative [26]. Central to critical theory is an understanding of the systems, structures, and social relations that (re)produce the marginalization and oppression of certain groups [27]. Illuminated in its social and historical context, critical theorists pay attention to how power is organized, wielded, and legitimized in society to perpetuate domination and social injustice in society [28]. Adopting a critical theoretical lens to the study of housing models in BC, we aim to explore and reveal how structures, systems, and relationships of power in the province perpetuate homelessness and housing precarity, and the injustices and adverse consequences associated with being denied the right to (adequate, affordable, suitable, and preferred varieties of) housing.

## Methodology

### Ethics statement

Research ethics approval for this study was granted by the Royal Roads University Research Ethics Committee. Informed consent to participate in the research was obtained verbally before participation.

### Reflexive thematic analytic methodology

The study explores the following research questions: (a) what are supportive housing tenants’ experiences with housing and related service providers in their current housing arrangements, and do tenants consider these experiences and arrangements to align well with the notion of consumer choice?; (b) how well do supportive housing tenants’ housing arrangements meet their own self-defined standard of acceptability and satisfaction; and (c) in what ways do these arrangements fit with notions of choice in HF and the supported/supportive housing literature? To explore these questions, we employed a reflexive thematic analytic approach [29–31], a well-known and highly influential qualitative research methodology, using data we generated from interviews with participants living in complex care housing and supportive housing in BC.

Reflexive thematic analysis offers tools to analyze various forms of data, including interview data, to develop “‘patterns’ (themes, categories) across cases” [[29] (p37)]. The reflexive approach to thematic analysis recognizes that “themes cannot exist separately from the researcher—they are generated by the researcher through data engagement mediated by all that they bring to this process (e.g., their research values, skills, experience and training)” [[29] (p39)]. Here, data coding, an inherently subjective process, is “unstructured and organic, with the potential for codes to evolve to capture the researcher’s deepening understanding of the data” [[29] (p39)]. The reflexive researcher is one who continuously reflects on their biases and assumptions, and how these influence, shape, and delimit their coding [29,30]. In undertaking this research, we applied, recursively, the six-phased approach outlined by Braun and Clarke [32], specifically: (a) familiarization with the data; (b) identifying codes; (c) generating initial themes; (d) reviewing and developing themes; (e) defining and naming themes; and (f) writing up the study.

To ensure our research is trustworthy, we included the following approaches for research rigour [see [30,33]. First, we engaged in regular peer debriefing sessions as a research team to discuss the data coding and analysis. Second, we sought to provide rich and detailed descriptions to provide a rich account of participants’ responses and narratives. Third, we infused processes for reflexivity throughout the research enterprise by recurrently reflecting upon our values, beliefs, assumptions, and positionalities and how these affect our interpretations of the data (we have documented these for the reader in the section below). Finally, before submitting the manuscript for publication, the research team presented the study findings to government officials in BC, allowing for broader take up of the research and bolstering its robustness through peer scrutiny of the findings [see [33]]. We applied Braun and Clarke’s [34] qualitative reporting guidelines to help structure our work.

### Reflexivity: Author positionalities and background of the research team

TS-C (first author) is a White, cis-gender woman, mother, and researcher who has spent the last two decades researching poverty and its related symptoms and outcomes (e.g., homelessness, food insecurity, intersectional inequities, disabling conditions, etc.). TS-C grew up in social housing in Toronto, and in a single mother led low-income household. She embraces a critical realist ontology and espouses the theoretical tenets of critical feminism.

HD-A (second author) is a White, cis-gender woman from Victoria, BC. She has a multi-disciplinary background, working in finance with a Bachelor of Commerce, though has a graduate degree in environmental studies, with research areas focused on social justice and welfare. As a bio-child in a foster family with a tenure of nearly two decades, she employs a trauma-informed approach to research and a social determinants of health lens.

DP (third author) is a White, cis-gender mother living in Toronto, Ontario. She grew up in Toronto Housing. During the coronavirus pandemic, she and her child lived in a violence against women shelter where she directly experienced aspects of the city’s application of a HF policy. She has worked in public health in British Columbia and Ontario, and primary care from Canada’s West Coast to the East Coast. She is currently pursuing a doctoral business administration degree exploring how to apply a systems approach to trauma-informed business practices.

CAM (fourth author) identifies as a White, cis-gender woman living in Southern Ontario. For over a decade, she has conducted research on intersections between poverty and mental health, with a primary focus on homelessness prevention. She worked for over a decade in shelters for individuals who experience homelessness and as an occupational therapist in a range of mental health services, including community mental health and the corrections and forensics systems. Poverty, mental illness, and addiction have touched her family life in a range of ways, and have come to influence her research and professional interests in these areas.

Prior to study commencement, the principal investigator ([PI]; TS-C), who has lived and research experience with the study population, hired and trained two research assistants (RAs) to assist with the study. Having lived experience of being unhoused, the first RA (DP) conducted the bulk of the research interviews. The second RA, hired because of their previous work experience in the supported housing sector, left the study due to other work commitments after having conducted only a few interviews. HD-A, with research experience on welfare and applying a social justice lens, was hired after the interviews had been completed and assisted with the data analysis and manuscript writing. CAM has worked with the PI on previous research projects and has published extensively on housing and homelessness issues in Canada. Significant time was spent as a research team drafting the ethics protocol, discussing how to build rapport and apply a trauma-informed interviewing approach with research participants (as outlined by Isobel [35]), and debriefing about the data analysis, created themes, and manuscript development.

### Sample, sampling, and recruitment

The research team conducted semi-structured one to one-and-a half-hour interviews with individuals in supportive housing and complex care housing. Colleagues at a government ministry provided the research team with referrals to housing managers and/or administrators responsible for provincially-administered housing facilities and/or services. The names of said facilities/services are undisclosed to ensure participant confidentiality and anonymity. A snowball sampling recruitment approach [36] was also employed in which tenants were asked to refer others in their apartment building or complex who might be interested in hearing about the research.

Research candidates interested in hearing more about the study were asked to contact DP (or the other RA hired at the time) by phone or email, and if they wished, to schedule an interview. At the interview, DP (and/or the other RA) reviewed the study information and informed consent letter before asking research candidates whether they would provide their verbal consent to participate. Interviews were conducted by phone or online, via Zoom. In recognition of their time and contributions to the study, participants received a $50 cash incentive. The interview schedule included a variety of questions designed to build rapport with the participant before asking them to provide a rich account of their housing experience (e.g., introducing the study, the researcher, and asking participants to talk a little about themselves, the kind of housing they lived in, and what the process was like acquiring their housing placement, etc.). The interview guide included questions, with subsequent probes, designed to prompt the participant to take the interviewer along a chronological account of their housing journey, from application, to intake, to their current day-to-day experience (e.g., regarding the location, affordability, adequacy, and suitability of their housing, and how they felt about their neighbours, housing service providers, and the housing-related programs they accessed).

The attending RA requested consent from each participant to either permit the interview to be recorded for accurate transcription, or should they prefer, for hand-written notes to be taken. All participants permitted the recording. Though the interviews were designed to be one-on-one, some participants opted to have a support person (e.g., a staff member) present during the interview. Such an approach is congruent with a trauma-informed approach to qualitative research and interviewing [35]. While we acknowledge that the presence of housing personnel may have impacted the participant’s responses, the safety (and comfort) of the participant was considered a priority.

Prior to the interview commencing, the RA informed the participants that they could choose not to answer any question they did not wish to answer and they could withdraw from the study at any time; they would still receive the $50 should they decide to withdraw (no one withdrew). Although in one case, a research participant experienced a mental health crisis during the interview, and DP thus turned off the recording to give the participant the time and space they needed in that moment. Clinical staff were available on-site and the participant was invited to return to the interview if and when they were ready to do so. In this instance, the participant wanted to continue and was able to resume later, thus ultimately completing the interview. All participants were provided with a list of community mental health resources at the conclusion of the interview should they have experienced any adverse reactions as result of their participation in the research. Once completed, the recordings of the interviews were transcribed verbatim through a professional transcription service and reviewed by HD-A and DP for accuracy. Pseudonyms were assigned to study participants to ensure their privacy and anonymity. TS-C, HD-A, and DP utilized NVivo (v. 14) qualitative software to organize, sort, and analyze the data. All authors participated in debriefing sessions to analyze the data, identify the themes, and prepare the manuscript.

## Analysis

We conducted interviews with 24 individuals (n = 6 in complex care housing; n = 18 in supportive housing). See S1 Table for a breakdown of participant demographics, across a number of characteristics. In our reflexive thematic analysis of the interview data, we constructed several themes that spoke directly or indirectly to dimensions of consumer choice, although the essence of these feature dimensions of ‘constrained choice’ for housing tenants in supportive housing and complex care housing in BC. The analysis outlined below takes up themes related constrained choice and feelings of gratitude for housing, characteristics of supported housing, costs of supportive housing, perceptions of physical and psychological safety, and perspectives on housing rules, compliance and punishment, and housing readiness.

### Constrained choice: Take it or you’ll ‘have nowhere to go’

The wait time to be assigned supportive housing ranged for participants from as little as a few days to as long as several years (note: there is one waitlist for supportive housing across BC, although units and/or buildings fall under the purview of the province, health authorities, or BC Housing delivered through non-profit providers). Before entering their supportive housing unit, some participants had been discharged from the hospital, were staying with friends or family, living rough on the streets, or ‘in a motorhome’. Overall, the data do not provide us with a clear description of when and how housing arrangements were selected and/or assigned for participants, as these processes or recollections varied significantly from one participant to the next. Jessica (single woman in supportive housing) was in the minority. For her, it only took a “couple of days” to get into housing. Jessica had “applied twice. The second time, I got in within 30 days”, because “basically, like, after a while your application runs out after I think a year.” The wait for Emma (single woman in supportive housing) was “a year and a half” but “probably two to three months” for Ryan (single man in supportive housing) from when he was “wanting to get out of the homeless/transitional (shelter) into a place.” Many stated that they had waited three to five years to be allocated housing. For Laura (single woman in supportive housing), it was significantly longer, as she described:

Well [laughs], that’s a tricky question. I had been on the (provincial) so-called waiting list for like 15 years trying to get into (building name). However, every time I rented a place, they didn’t bother to tell me that they take you off the list [laughs]. So, anyway, it’s a long time.

Laura went on to say, “I’ve signed up for BC Housing as well, but the waitlists are like four years long.” It helped to have someone who could advocate on your behalf, as Lisa (single mother living with her child in supportive housing) shared:

I kept calling every week to see if they had space available… I started to get very discouraged because I wasn’t hearing anything and I had to stay in the shelter for Christmas, which really sucked. And then they’re like, ‘Well, we’ll start advocating for you.’ So, the people at the shelter advocated for me to get into here, and then I got a phone call – what was it, January 24th, telling me that they wanted to do an interview process to see if I was a good fit for this place. And then by June 26th, I had my rent and my damage deposit and I was allowed to move in.

Paradoxically, many participants acknowledged that they were satisfied with their living arrangements even though they did not feel they were given a choice about the supportive housing unit/building they were assigned to. This was demonstrated in the exchange below between the interviewer and Ray (single man living in supportive housing):

Ray: They showed me... I think they did, because at that point, right, I was also kind of told that if I didn’t take it, I’d probably have nowhere to go…

Interviewer: It was kind of that or nothing?

Ray: Yeah, well, a roof is a roof, right? At the end of the day… It’s not for everybody, if you know what I mean. You need a certain level of survival skills, I guess.

Some participants, like Lisa, “had hoped for a two-bedroom” apartment, while others wished for an alternative facility better suited to their needs and preferences, like Kyle (man living alone in supportive housing) who “would have rather been at (building name) with [his] dad. He’s got some medical issues.” Similarly, Laura indicated that she “(w)ould rather be over in (name of building). It’s more for seniors and people on disability, and there’s not these restrictions and wellness checks. I’m disabled, but I’m not a drug addict or an alcoholic or anything like that.”

Jason (single man in complex care housing) expressed, “I was very desperate for a home. As I was homeless for several years. And so, I was ready to just take whatever was available and this came up here.” Likewise, Dawn (woman living alone in supportive housing) remarked, “There’s (sic) no choices really, because there’s not much available, right?” Consequently, the ‘choice’ presented to most participants was limited to only two options: take the unit offered or become, or remain, homeless.

Consumer choice is constrained by limitations in housing stock, specifically the number and type of units vacant at any given time, which are not necessarily responsive to what participants want or need at the time of allocation. Lisa shared:

Lisa: Yes, some have three, some have two (bedrooms), some have one and some are bachelors. It’s just kind of pick of the litter in what they have available at the time.

Interviewer: Just whatever comes up… Okay, so you weren’t asked about your preference of unit?

Lisa: No.

For some participants, like Mary (single woman living in supportive housing), it was not the unit itself but the geographic location that mattered most. Mary stated, “I didn’t care... it was downtown... (so I) said, ‘Yeah.’” Gary (man living alone in supportive housing) also felt compelled to accept an assigned housing arrangement to ensure he stayed in the city he had been living in, “because this was the only place they had available in (city).” Gary admitted that he “would rather be somewhere else.” Consigned to little choice, it was, at the end of the day, “an opportunity for a roof over your head and safety”, as Mary stated.

### Amid rampant homelessness, I’m ‘very blessed to be here’

Despite not always being assigned their first choice in housing arrangement, many participants expressed their deep gratitude for being placed in safe and affordable housing, recognizing that the alternative, invariably, was homelessness. Anne (woman who lived alone in supportive housing) shared that she “didn’t ask (about the housing assignment), I was just so happy to just to get a place to live.” Similarly, Mary commented that she felt “very blessed to be here.” The following exchange captures Leanne’s (single woman in supportive housing) gratitude.

Leanne: Yeah. I didn’t have a second choice just because of – it was either that or I had to move in with family out of town, because I had nowhere to live.

Interviewer: So… it sounds like one of your only options or one of very few options.

Leanne: Yeah. So, grateful. So very grateful.

Similarly, Megan (woman in complex care housing) shared:

I’m just really grateful to have my own apartment here. I try my best to make the best of it and. You know, it’s really [a] funny place. It makes me laugh here. Just something about it. It’s a lot of character. Just the people that live here and the staff and the things that happen… I’m happy to be here.

### Characteristics of supportive housing

Some of the buildings that participants lived in were brand new, others were quite dated. Michael (man living alone in an updated complex care building circa ~1990) shared that because “it’s a modern building, it’s well, you know, [has] nice hard work floors.” Michael also characterized his building as “beautiful” even while “there’s work outside that needs to be done.” Yet, despite the age of their buildings, participants noted specific issues that could be rectified. Mary disliked that “there is no place for a common room to play cards, to talk, to do a puzzle. And so, you’re stuck making friends with your neighbours and going into their space, which I’m not really into that, that means I have to clean my place every damn time, hey, so I’m not into it.”

Not all buildings, as some participants noted, were designed to be fully accessible for occupants. Michael, for example, “had stairs and I didn’t like the stairs. Being 120lbs overweight it’s really hard for me to take the stairs all the time.” Dwayne (man living alone in complex care housing) had a scooter but couldn’t “bring it upstairs, [because] there is no elevator here, right?” Tanya, who lived in a building constructed around 2020, similarly commented:

There is (sic) only stairs, and I mean for anyone going up and down the stairs, if they’re elderly or young, they could accidentally mess up their knee. And I’ve had issues with my knees going up and down those stairs.

Given the absence of an elevator, one facility designated the first floor for people with mobility issues. Amanda (single woman in supportive housing) explained:

They have a wheelchair ramp and – there’s no elevator, mind you, but they have the first floor designated for people that have mobility issues. So, they have all that in effect. Yes, there’s a safety ramp.

However, as Ryan (single man living in a supportive housing building built around 2020) expressed, property management cannot put everyone with (current and potential) mobility issues on the first floor.

I’m glad they put me on the second floor because I have back issues myself and pretty soon – like [I will need an] elevator… there’s going to be a lot of us that have, you know, issues like that that they can’t put us all on the first floor if there’s only 10 units on the first floor… they need to make entrances more accessible. In the front door, the ramp is up to the side, and the back ramp is not very good… there’s a door that opens to the stairs, and it blocks the stairs.

The lack of an elevator notwithstanding, Lisa (in an accessible unit) shared:

Yeah, you’ve got elevators for people who have diseases. I’m in a disability unit, so I have a walk-in shower, there’s bars everywhere for me to grab onto… I don’t have anything below my waist that I have to worry about bending over to access. So, it’s all, like, wheelchair accessible, and yeah.

Mary felt that the building could have been better designed for wheelchair accessibility:

So… out of 28 places there are only two that are wheelchair accessible, so they could easily have designed a few more of those… there’s so many people with scooters nowadays and also walkers. And so, none of these places are adaptable to, if I need mobility, I have to move down the hall or out, there’s no transition here… And so, these things aren’t flexible, they’re just made bingaty-bang, bangaty-boom, here you go.

Although there were a few participants who felt that their supportive housing building was not close to local amenities, others were situated in the downtown core where tenants had easy access to a range of shops and services.

Interviewer: Is your housing in a convenient spot with easy access to services that you need?

Jessica: That would be a five (out of a scale from 1 to 5, with 5 being the highest).

Interviewer: That’s a five, so really good access.

Jessica: Yes. We’re right downtown, close to everything.

Caelyn (woman living alone in supportive housing built around 2020) shared:

Well, there’s Safeway (supermarket) and Shoppers (pharmacy) nearby, but Superstore and Walmart are the cheapest places... for any sort of like, say like furniture or decorations, like Winners, big box outlets, those aren’t really very close... but for services like (outreach program) and (program), and like banks and like the welfare office and that, so I’d probably give it a 5 (out of 5, with 5 being highest) to those kind of things.

It was the same for Michael who shared, “Yeah, because you’ve got the fire department, you’ve got the counseling, you’ve got shopping, buses, a doctor’s office. Like, it’s all within, like, I don’t know, two blocks.” Being outside the city, where Jason is, is “where it kind of becomes slightly difficult because it is out of the city center where I like to be. So, it provides a bit of a barrier being out of town as it is.”

Not having sufficient space in their unit was a recurring theme for many participants. While some, like Emma (in a building built around 2015), who “love(d) small spaces”, were in a bachelor apartment, others, like Lisa (in a unit built roughly in 2015), “would like to have (their) own bedroom, and... could use some more storage [space].” Likewise, Tanya remarked:

I honestly don’t have enough room. But I mean I’m not picky about like my living arrangements over where I’m staying. But I’ve got the smallest unit, they said. And I took it right away because I knew I needed somewhere to go and somewhere to live.

Kyle’s (supportive housing, built around 2020) building is not equipped with an oven, likely given concerns over safety, but this impacts his ability to cook his own meals.

Yes, there’s enough room. It would be nice if they had ovens. But I can see why they don’t. You know, because there’s some people here that aren’t all – they think different, so I think they might start a fire or something.

Other times, the design and/or arrangement of the rooms in the unit could be peculiar. Shona (single woman in complex care housing built around the 1990s) commented, “Oh yeah, so in the units they sometimes have the bedroom locked off so that the bed is in the living room space.” In one supportive housing building Ryan described “there’s no rent there, it’s free, and there’s pods they give you. There’s no rooms, it’s just like cubicles.”

Consumer choice is not only important when deciding upon a supportive housing arrangement, but also in decisions related to remaining in one, particularly when considering issues related to accessibility, adequacy, suitability, and safety. A few people commented that their building needed renovations and/or repairs, particularly older stock. The heating in Mary’s unit was deficient, making her feel chronically cold.

The heating is crap, they have one heating thing at the far end, and there’s no other way for air to get heated, except if you’re at the far end opposite the kitchen. There’s no heating in the bathroom. It’s like, ‘Oh my God, what is going on in this place?’ It’s like living underground, I’m above the garage, it’s totally unsuitable, it’s cold, if I had a deep, deep pocket, I’d have heated floors, but you can’t make any changes.

Some ranked the adequacy of the building extremely high (5 on the scale from 1-5), and Laura (in a unit built around 2020) indicated that “it’s in good shape. They check every month the smoke detectors, and they inspect the rooms and ask you every month if there is anything that needs fixing.” Ryan, however, felt that the inspections weren’t always taken seriously.

(Things) were missing when I moved in here... I didn’t know if it was cleaned or not, but there’s stains on the floor, damage to the unit, and none of that was repaired before I moved in. They do do – one of the services I forgot to mention, they do do room inspections once a month. Put a thing on your door to let them know you’re coming in. But it isn’t really much of an inspection. I’ve been sleeping before when they did it and I didn’t wake up. They pretty much just stick their head in the door and make sure you’re alive.

Tanya shared that

Because there are like holes in the ceiling… like, it’s broken down. So, like you can see part of the wood that’s like above in the ceiling in the common room, and that’s one spot there. They’re probably trying to fix that or figure that out, I’m not 100 percent sure... Because that could be a little bit dangerous if someone might fall through the floor.

Yet when there are items that require attention or fixing, these can take considerable time.

And my one friend, her whole – it’s like all water damaged from a leak that she’s told them about and they haven’t come in to do anything. And it’s going to cause by now serious damage… and there’s mould and they’re still having her in her apartment. And she’s like, ‘What do I do? I’ve asked them and asked them and asked them but they’re not doing anything’ (Amanda).

Mary similarly expressed:

I’m not quite sure how long you’re supposed to wait these days when you need your fob door opening, are you supposed to wait six months?... We all kind of lowered our standards for acceptability around timing and how long things could take.

### Reasonable housing costs

Participants largely felt that their rental costs in their housing arrangements were reasonable and affordable, particularly in the context of soaring private market rental costs. These were calculated, according to Jessica, as being 30 percent of a tenant’s overall income. As such, the rental costs, for a bachelor apartment, tended to be roughly $375 to $570 a month (a far cry from the average $2,087 monthly rent in BC in December 2023, the highest across the country; [37]). Ryan also indicated that additional funding was headed his way, “because it’s getting so expensive with groceries and the rent... they’re raising it $125 in August which isn’t enough, but good enough to offset the price in place.” Many had their rent costs taken directly from their social assistance benefits, with the remaining funds to attend to their food and other necessities. For Dawn (living in supportive housing built around 2020), it was “the least rent I ever paid, ever.”

While the subsidized fee structure was valuable in helping tenants afford their monthly rental costs, they still struggled to make ends meet daily. Tanya shared:

I never really have enough for food at the grocery store to buy there. And it seems like I’m running low on food. So that’s why I’m reaching out to get a food hamper almost every month... I just have trouble saving money for food and going to the grocery store. I have rent to pay, and a phone bill, and minutes...

Thus, Kyle found that the monthly budget was simply ‘never enough’ to cover everything.

### Mixed perceptions of physical and psychological safety

Participants had mixed perceptions about their safety while living in supportive housing environments. While a few claimed that their building was “pretty secure... there’s not much like loudness that goes on there. And pretty good, like, people that live there.” For Emma, it was the lack of safety within the city itself, not specifically the building, that was concerning.

Well, it’s not necessarily like the building itself, it’s just the part of town that we’re in. But that’s (city) right now. Like, I had to call the RCMP [Royal Canadian Mounted Police] on a man outside yesterday who was smoking crack in the parking lot sort of thing, right, and like lingering around the building. Well, I was worried he was going to overdose too.

Emma also shared concerns over her and other tenants’ psychological safety.

I wouldn’t want to be, like, outside late at night here very much kind of thing. But, like, the building itself, yes, but, like, psychological is a bit of a different story, I think.

This was also the case for Ryan, who was struggling to remain sober even though there were ‘still a lot of drugs… circulating the building’.

It’s a mixed bag, right? I have kids that I’m actively involved with or whatever and so sobriety is obviously something that I’m struggling to do and aiming for... I’m out of the downtown core but there’s still a lot of drugs circulating the building. Even people like dealers... So it’s just hard when people knock on your door in the night, want you to score for them, or even during the day... And so I really had to sort of barricade my door in just between work and here and my kids, my decision, is really – I don’t even answer my knocks half the time because it’s just probably something that I don’t – that isn’t healthy to interact with.

Certain areas of the building can feature greater safety than others, as Amanda noted:

… On the third floor they can’t hear what’s going on so it’s not always safe up there. And even on my floor, on the second floor, like I’ve had to call them many times saying, ‘There’s going to be a fight breaking out, you better get up here,’ yes or, ‘I’m concerned for this person’.

Being exposed to traumatizing experiences can contribute to occupants’ sense of psychological safety, particularly if these are profoundly unsettling and/or frequent. Lisa shared a harrowing example detailing one of their experiences:

Like the one girl came out of her room and she’s standing there with like six knives in her hand not knowing where she is or what’s going on. And so we had to call the staff up there, and it’s like yes, she’s just clear out of her mind. She wasn’t aggressive or anything but she’s just really schizophrenic...

Not being welcomed in the community can also take a toll on individuals’ psychological safety and wellbeing. Ryan shared, “We have the fire truck here at least once a week, sometimes more, there’s so much smoking in their units. We’re not despised, but we’re not liked in the community, right?” Ray might have reconsidered accepting his unit given his perception that the building was “an addict’s paradise” as evidenced in the following exchange:

Ray: If I had known more about it, then maybe I’d reconsider.

Interviewer: Okay, why’s that?

Ray: It’s basically an addict’s paradise, if you know what I mean?

Interviewer: Okay, so lots of substance use?

Ray: Yes.

Rather than providing a cozy and comfortable environment, Kyle felt that living in the building was ‘like preparing for jail or something’; he expanded:

Kyle: It’s like preparing you for jail or something, I don’t know.

Interviewer: Okay, why do you say that?

Kyle: Doors slamming and – all the time and –

Interviewer: It’s noisy?

Kyle: Noisy, yes, gates, and just the rules and...

A concern was also raised about the limited soundproofing in Tanya’s building.

Oh, the way the building is built. It echoes a lot through the building. So anytime people are in the hallway, neighbours will obviously hear what they’re talking about and what they’re discussing necessarily, even if it’s supposed to be a private matter.

### The rules: Some stupid, some reasonable

Participants described various by-laws, policies, or rules applied in their supportive housing buildings, some deemed helpful, others apparently less so. Tanya commented:

And a lot of folks have loud voices in the daytime. That’s okay. But in the evening, just be cautious of the loudness, for the by-law law. And understand when it comes down to it, that that’s a rule. You don’t want to go over that rule…

In contrast, Jessica deemed, in the exchange below, that the rules of her building were helpful, particularly in promoting greater security for tenants:

Interviewer: What kind of rules are there?

Jessica: In our building, there’s no men or significant others allowed. The children have to be monitored at all times and your key is your key. You’re not allowed to give it to anybody else. The security at this building is amazing.

Interviewer: And so, do you find these rules helpful to you?

Jessica: Absolutely.

Caelyn commented that the “guest rules are kind of like the annoying, like, most annoying rules.” Some participants described having visiting hours for their guests that adversely affected their ability to be hospitable to family and friends. Laura, for example, begrudged the fact that she could not have her daughter and grandchildren overnight when they came to visit from out of town:

Yes, there is (sic) rules... like, our visiting hours are from 10:00am to 7:00pm. I can’t have – like, I have a daughter and grandchildren so if they came to town, I wouldn’t be able to have them overnight... like, they don’t have those type of rules over at the other place I was trying to get into.

Amanda’s building did not allow visitors at all after the COVID-19 pandemic.

I understand at the beginning there we signed our tenancy agreement about the visitors, it being COVID and all, that we wouldn’t have visitors right away... But then as soon as that died down, we would be able to have our visitors. Well, so no, we’re still not allowed visitors in our units at all and I’m not quite sure why.

Furthermore, Emma shared that “you have to stay with your guests the entire time they’re here. So, you have to walk them to and from the door. No problem with that... You have to attend a weekly, like, floor meeting. No problem with that either.”

Several participants noted having to comply with regular inspections and/or check-ins as part of their occupancy in the building. Jessica noted, “Once every three months, we do an inspection and every day we have to check in... or else they come check on us.” For some, there were overt or tacit rules governing tenant behaviours, including how they should treat others in the building. Emma shared:

It’s pretty lax, and it’s basically just the usual stuff, be respectful towards people or you could get evicted kind of thing. Your substance use can’t affect others in the building, that sort of thing. So, no, I think they’re all... very reasonable rules.

Amanda described the power imbalance in supportive housing, whereby tenants have little say about the rules and their enactment, while housing administrators “can change the rules, our tenancy agreement, they can do it whenever they want. They can change any aspect of that without notifying us and anything is subject to change without notice.” Amanda continued, noting the arbitrary nature of rules’ enforcement in some buildings,

Basically, they can kick us out whenever they feel like it and actually take our key cards away and say, ‘Okay, you’re kicked out for two days, then you can come back’.

Some participants felt that the rules imposed were reasonable and contributed to a “really quiet environment”. Amanda stated, “Yeah, no fires on the patios and stuff like that, just typical, no laundry flapping and no dogs, no barking, and so it makes it a really quiet environment.” Craig (single man in complex care housing) also claimed, “Rules and guidelines are helpful because they maintain a good, healthy, living environment.” For others, like Anne, the rules were deemed to be “very strict, extremely strict.” She went on to say:

We’re all alone, single tenants, and not one – we’re not allowed to have company like stay overs... You can’t hang pictures on the wall, you can’t use nails. If you use a thumbtack and it makes too big a hole you could charged for it. It’s just hideous.

Laura felt the rules were

(G)ood for the most part, (although) they do have double standards here. There’s favouritism. Not everybody gets the same rules, it seems. But we all deal with it.

Ray believed that some rules made sense, while others were ‘stupid’ and prone to change with little notice.

Ray: Certain rules, yes. Yes, I can see the necessity of some of them but... I think it might be privately owned, so there’s usually some weird bias rules that come up that don’t last long because they’re pretty – what’s the word? Stupid.

Interviewer: So, it sounds like maybe the rules change pretty frequently.

Ray: Yes.

Several participants were troubled that they could no longer care for their pet(s) while in their supportive housing building, even though these are cherished and known to have therapeutic value. Anne noted:

Anne: Oh, my pet died, thank God.

Interviewer: Oh, I’m so sorry.

Anne: And other people in the building that had pets had to give them up.

Interviewer: Oh, that’s so sad.

Anne: And it broke (my) heart... It’s very heartless.

Emma similarly remarked:

Emma: I think subsidized housing should allow pets. I’m going to put that on the record.

Interviewer: That you feel they should allow pets.

Emma: Yes, I miss my cats... and pets are very therapeutic, right?

Interviewer: Yes, I completely agree.

Indeed, people working through mental health challenges and their sobriety can, as Tyler claimed, be ‘more at peace’ with a pet.

Sobriety, and just my mental health, right. I get anxiety being around most people, and you know, people are rude and disrespectful, and that affects your mental health, right, because people will say sh** or they’ll poke – you know... I realize I feel so much better and I feel more at peace when it’s just me and my cat.

Anne suggested that pets are what keep some people alive.

The one woman [in my building] did have a pet and had to give it up because she was in a wheelchair and moved into the building. And for those people, my heart goes out to them because that’s what kept them alive. And now there’s nothing to keep them alive.

### Perceptions of compliance and punishment

A few participants, like Michael, felt that the tenants in their buildings were subject to punishment if they did not readily comply with the directives of supportive housing service providers or administrators.

You know, there’s favouritism. And there’s compliance, non-compliance. And punishment. Yeah. I’ve been on two citations already. Right. And I found it unnecessary actually. Because it had nothing to do with me being violent or anything, right, or having me cause any mayhem. I just think management are (sic) trying to make decisions.

Lisa felt that to remain in her unit, she “need[ed] to try to refrain from drugs and alcohol.” Leanne similarly noted, “No males here. And no drinking, no partying, and that kind of thing, you know.” Lisa further shared that the weekly meetings with her worker and residential floor were mandatory conditions placed on her occupancy.

And then, you know, you do your one-on-one meetings every week with a worker. And you do your residential floor meetings every week. And those are the only two mandatories in order to stay here.

The penalties for disregarding the rules could, according to some, be severe. Lisa shared, “Yeah. The biggest one is not to have any males on the property over the age of 18... And that’s an immediate eviction.” At the same time, some participants, like Ryan, wished to see further disciplinary actions taken against tenants that are acting ‘really bad’.

Even though there is no additional bias and there’s fairness, still some people get a lesser degree of severity. But there’s people here that should be on clear disciplinary effects, right? Like... if you’re really bad, they kick you out for a number of days and stuff like that... But I’ve witnessed things... it’s been repeat, or there’s been interactions I’ve witnessed and the person should have at least... be under that kind of consideration. Because they haven’t received any kind of – and it’s just like abuse to the staff and all sorts of stuff like that.

### Internalized readiness requirements and housing disparate groups in a market marked by scarcity

Participants were concerned about the short supply of supportive housing in the province. Jessica commented, “They need to make a lot more buildings like this... We need a whole bunch more.” Other participants made specific recommendations for certain populations. These, however, were often contextualized within a readiness (stepped or phased) transitional paradigm. Emma stated:

I really think with, like, second stage supportive housing there needs to be a lot more for women in this province. We’re lucky to have another one getting built at (city), but when I was looking, like province-wide, there’s hardly anything. I also feel very strongly that there needs to be more housing for people in active addiction, with harm reduction being a short-term goal and recovery being a long-term goal.

Similarly, Ryan shared:

They need to have more second stage programs after treatment or even without treatment or second stage. And like [the] halfway house thing where you live in a house, then you have to be actively seeking employment, you have to stay sober, and stuff like that.

Ryan went on to say:

We need more second-stage housing... and even third stage, whatever that looks like. Basically, a whole remapping or revamping of the housing system where they actually have places like more supports like this for people who are trying to stay clean, who are sober or trying to remain sober...

The expectation of housing readiness was apparent in these statements, and in one by Amanda, who commented that there are “now so there’s two (facilities) – there’s the (building name) where it’s supposed to the stepping-stone in order to get into my housing, into (building name) where I live.”

Ryan felt that there were few supportive housing arrangements available for men, both those fleeing abuse and those with children.

And they’re actually putting up a second one (building), which is too bad because we need one for men too. Not necessarily fleeing abuse, although it does happen, but just men with kids that are in housing parameters that they can’t fill, they need to have one…

Anne, meanwhile, felt that we need to “get more housing for seniors,” while Dawn asked, “Why isn’t there supportive housing for two people?”

It kind of depends on their needs actually... Like for me, a single person, it’s totally awesome, but if you have like – for parents with kids and stuff like that, it might not be totally the best just because they don’t always get the space that they need. So, I would recommend it if you, like, are desperate though, right? Of course. (Dawn)

## Discussion

This reflexive thematic analysis developed several themes related to consumer choice in supportive housing programs in BC (for themes and exemplar quotes, see S1 Data), including the essence of the data having to do with ‘constrained choice: Take it or you’ll ‘have nowhere to go’. Paradoxically, while participants in the study felt tremendously ‘blessed’ and grateful to be housed, they often were not extended considerable choice in their building or housing location. Some described being in housing units that were inaccessible (e.g., no elevator access), unsuitable (e.g., lacking sufficient space or in buildings needing repair), or governed by (explicit or implicit) rules (e.g., about pets, visitors, individual behaviours, expectations regarding abstinence and/or sobriety). The essence of constrained choice in these data, and the associated themes constructed above, emphasize that while HF has been adopted as an approach by supportive housing and complex care housing programs in BC, the underlying components of consumer choice and self-determination may be infrequently practiced in these contexts.

Consumer choice and self-determination are central pillars of HF principles [38]. Similarly, the cornerstones of supported housing include choice, autonomy, and normality [18]. Parkinson et al. [19] note the differences between custodial, supportive, and supported housing models. *Custodial housing* reflects the value of custodial care, *supportive housing* (a label employed by the authors, not referring specifically to BC Housing’s supportive housing program), underscores the value of rehabilitation, and *supported housing* espouses the value of empowerment and community integration. The model of custodial housing offers little choice over housing to patients/clients and maintains restrictive rules and staff control; supportive housing provides some choice and some rules for residents, and shared control; while supported housing reflects considerable choice, few to no rules, and tenant/citizen control [19]. To date, few studies have examined the dimensions of consumer choice, and how these are perceived and experienced by tenants in supportive programs that ascribe to the tenets of HF [20]. Our study therefore provides unique insight on how consumer choice is understood and enacted in supportive housing arrangements in BC.

Core housing need, according to Canadian Census-based and National Housing Strategy-based housing indicators and data, is defined in terms of meeting *adequacy* (i.e., not in need of major repairs), *suitability* (i.e., sufficient space for the make-up and size of the household), and *affordability* (i.e., housing costs below 30 percent of before-tax household income) standards [39]. Participants in our study noted that their housing arrangements did exceptionally well in terms of affordability, given that their rental costs were calculated based on 30 percent of their household income and were thus well below what one could expect to afford in the private housing market, particularly in BC where rents have steadily increased [40]. Many in our sample were also satisfied with the adequacy of their units, largely because their buildings were quite newly built and had not yet had time to deteriorate and did not require significant repair. Suitability, however, tended to be an issue, as some participants felt that they ‘honestly don’t have enough room’ given the small size of the rooms and/or units, many featuring little to no storage space. While these indicators help us explore whether people’s core housing needs are fulfilled, there are certainly other dimensions of consumer choice of importance.

Study participants discussed preferences related to neighbourhood and community characteristics. Participants favoured buildings located in urban centres, close to necessary services and amenities, over ones situated in suburban or rural settings where access to these can be limited. Gonzalez and Andvig [41] suggest that consumer choice related to neighbourhood and community context cannot be underestimated. In their meta-synthesis of qualitative research on tenants with serious mental illness, the authors found that housing contexts characterized as ‘mental health ghettoes’ were deemed to be unsuccessful, as such environments induce nervousness and fear, increase stigma and unfavourable attitudes towards people with mental illness, and promote poor community integration and networking. In such contexts, people do not feel like they ‘fit in’, belong, and are often forced to cope with discrimination and devaluation; all of which have detrimental impacts on tenants. Wright and Kloos [42] similarly found that neighbourhood variables, particularly those relating to the social environment, may be more important predictors of well-being than physical housing characteristics, and thus ones to remain attentive to.

In programs with high alignment with supported housing principles, tenants have choice over both their unit, and its location [15]. As such, the mixed perceptions of psychological safety noted in our findings may have important policy and planning implications for private supportive housing operators and governmental housing and/or health authorities, including the BC government. Moreover, references to the presence of significant rules, both reasonable or stupid, may have repercussions on tenants’ freedom to make their own choices. Piat et al. [18] argue for greater freedom and more lenient housing regulations after concluding that programs in which tenants are empowered to make free choices on matters of importance to them, including substance use and the care of pets, can help foster a sense of control over their living environment, and facilitate a sense of ownership and of being ‘at home’. As such, Padgett et al. [[43] (p82)] suggest that “program philosophies favouring choice over restrictions and empowerment over compliance deserve consideration as not only effective but humane.”

Several study participants made recommendations about extending supportive housing through HF to specific groups, albeit these largely fit within the confines of a TF or readiness paradigm. Here, it appears that people who have experienced housing insecurity and/or homelessness may have internalized expectations about how one should act and/or behave to demonstrate one’s fitness for housing. These also came through in the theme about compliance and punishment. Grainger [44] cautions us about the use of ‘soft’ measures in the contractual supportive housing lease that seek to redirect tenants’ thoughts and behaviours (see also [45]). Rather than adopting strict repressive tactics of control, case managers and service providers can often apply a series of nudges (in contrast to the apparent pushes of TF) to reform tenants’ subjectivities and internal motivations towards becoming obedient and responsible private tenants [23]. This hybrid mode of governance, to which Löfstrand and Juhila [23] speak, serves to naturalize and legitimize neoliberal logics, with their inherent social pathologies and inequalities. The authors thus question whether HF programs are not comparable to TF ones, reflecting a penchant for behavioural modification, when tenants are perceived to make questionable choices, given that “making ‘wrong’ choices (e.g., choosing not to pay rent or resisting the PHF [Pathways HF] teams’ home visits) is represented as individual ‘failures’. These are tolerated, and regarded as natural to some extent, but *repeated* ‘failures’ means that the choice-making of clients is curtailed” [[23] (p62)]. Repeated ‘failures’ may therefore jeopardize clients’ tenure in the HF model. Pleace [46], however, suggests that such an interpretation is incorrect, if not in part, as HF has distinct assumptions and impulses that differ widely from TF models. TF focuses on control, whereas HF emphasizes choice. In so doing, HF upends the neoliberal narrative of chronic homelessness that identifies the locus of chronic homelessness within the individual, and thus as a problem to be solved through the application of behavioural modification techniques. HF, rather, is extended with “tolerant persistence” [[46] (p334)] to people who even after several years may still use alcohol or substances and/or be mentally ill. Data that suggest that people must behave in preferred ways to enter or maintain their housing (e.g., demonstrations of sobriety, mandatory meetings with workers, etc.) deviate from consumer choice and from the harm reduction principles that underpin HF.

Given processes of neoliberalization and financialization in the Canadian housing market today [24], the expectations and perceptions of consumer choice of our study participants appeared constrained. Many were largely satisfied with their housing arrangements largely owing to the fact that they had a roof over their heads that they could afford (while so many others do not), with programs and support services that they could tap into should they so choose. Yet, limitations placed on tenants’ free choice-making related to behavioural nudges (e.g., regarding substance use practices) or housing regulations (e.g., limiting guests, pets, etc.) suggest that choice in these housing programs may not be as broad and expansive as it could be. Viewed through Parkinson et al.’s [19] framework, these may reflect a supportive housing approach (one, recall, featuring some choice, some rules, and shared control, underscoring the values of rehabilitation) midway on the continuum between custodial housing (strict rules, staff control, valuing custodial care) and supported housing (considerable choice, few to no rules, tenant control, espousing empowerment and community integration). Efforts then should focus on orienting complex care and supportive housing models in BC toward the dimension of supported housing on Parkinson et al.’s [19] continuum. This would involve removing housing regulations and rules that do not reinforce tenants’ self-determination, discarding behavioural nudges influencing tenants’ ability to choose their own treatment and/or services options, and considering changes to reduce or remove environmental stressors that might adversely impact tenants’ physical and psychological safety, applying a trauma-informed design lens [47].

### Study limitations

As this is qualitative research the findings described are not generalizable but could potentially be transferrable to other research contexts and settings (i.e., to supportive housing programs outside BC). Although consonant with a trauma-informed approach to interviewing, we recognize that having a housing worker or staff person during a few of the interviews could have influenced participants’ responses. Moreover, conducting interviews on the phone and/or online can make establishing rapport more difficult and/or conceal non-verbal cues that could provide greater contextual information.

## Conclusion

Housing models that encourage tenants to take an active and central role in the decision-making processes that affect them (from placement to continued housing retention) are likely to yield better outcomes, including improvements in their health and wellbeing [18,48,49]. Future research should explore how choice is understood, operationalized, and experienced from the perspective of supported/supportive housing workers, administrators, and support services providers in BC.

## Supporting information

S1 Table Participant demographics. (DOCX)

S1 Data Supplementary table with themes and exemplar quotes. (DOCX)
