# Supplementary material for: Exploring the bounds of consumer choice in supported housing: A reflexive thematic analysis of data generated from supportive housing tenants in British Columbia, Canada
Source: PLOS Ment Health. 2025 Dec 15;2(12):e0000505. doi: 10.1371/journal.pmen.0000505 (PMC12798549; doi:10.1371/journal.pmen.0000505)
Supplement: S1 Table — (DOCX) [file pmen.0000505.s001.docx]

**S1 Table: Participant Demographics**

Total Participants: 26

| **Housing Type** | |
| --- | --- |
| Supportive | 20 |
| Complex Care | 6 |
| **Gender (self-identified)** | |
| Man | 11 |
| Woman | 15 |
| **Age** | |
| 19-25 | 1 |
| 26-40 | 14 |
| 41-55 | 6 |
| 56-70 | 2 |
| 71+ | 2 |
| Did not specify | 1 |
| **Ethnicity** | |
| Caucasian | 14 |
| Indigenous and Caucasian | 3 |
| Indigenous | 6 |
| Did not specify | 3 |
| **Household** | |
| Alone | 22 |
| Alone with pet | 2 |
| Single parent with child | 2 |
