# Supplementary material for: Exploring the bounds of consumer choice in supported housing: A reflexive thematic analysis of data generated from supportive housing tenants in British Columbia, Canada
Source: PLOS Ment Health. 2025 Dec 15;2(12):e0000505. doi: 10.1371/journal.pmen.0000505 (PMC12798549; doi:10.1371/journal.pmen.0000505)
Supplement: S1 Data — (DOCX) [file pmen.0000505.s002.docx]

| **Theme** | **Exemplar Quotes** |
| --- | --- |
| Constrained choice: Take it or you’ll ‘have nowhere to go’ | Respondent [R]: They showed me...I think they did, because at that point, right, I was also kind of told that if I didn’t take it, I’d probably have nowhere to go…  Interviewer [I]: It was kind of that or nothing?  R: Yeah. |
|  | Well, a roof is a roof, right? At the end of the day. |
|  | it’s not for everybody, if you know what I mean. You need a certain level of survival skills, I guess. |
|  | hoped for a two-bedroom |
|  | would have rather been at (building name) with [their] dad. He’s got some medical issues. |
|  | (W)ould rather be over in... (building name). It’s more for seniors and people on disability, and there’s not these restrictions and wellness checks. I’m disabled, but I’m not a drug addict or an alcoholic or anything like that. |
|  | I was very desperate for a home. As I was homeless for several years. And so, I was ready to just take whatever was available and this came up and here. |
|  | There’s (sic) no choices really, because there’s not much available, right? |
|  | R: Yes, some have three, some have two, some have one and some are bachelors. It’s just kind of pick of the litter in what they have available at the time.  I: Just whatever comes up…Okay, so you weren't asked about your preference of unit.  R: No. |
|  | I didn’t care... it was downtown... (so I) said, ‘Yeah.’ |
|  | this was the only place they had available in (location) |
|  | would rather be somewhere else |
|  | It was an opportunity for a roof over your head and safety. |
| Amid rampant homelessness: I’m ‘very blessed to be here’ | didn’t ask (about their housing assignment), I was just so happy to just to get a place to live. |
|  | very blessed to be here |
|  | R: Yeah. I didn’t have a second choice just because of – it was either that or I had to move in with family out of town, because I had nowhere to live.  I: …So it was kind of – well, it sounds like one of your only options or one of very few options.  R: Yeah. So, grateful. So very grateful. |
|  | So you know what?... I have everything, I'm content. No, I'm just thankful I have a roof over my head, you know. It’s so hard to get a place, that’s all that matters. |
|  | I'm just really grateful to have my own apartment here. I try my best to make the best of it and. You know, it's really [a] funny place. It makes me laugh here. Just something about it. It’s a lot of character. Just the people that live here and the staff and the things that happen… I'm happy to be here... |
| Characteristics of supportive housing | first time…we’ve actually had built-in air conditioners in our place. |
|  | it's a modern building, it's, well, you know, [has] nice hard work floors. |
|  | But it is beautiful. There's work outside that needs to be done. |
|  | Oh, the way the building is built. It echoes a lot through the building. So anytime people are in the hallway, neighbours will obviously hear what they’re talking about and what they’re discussing, necessarily, even if it’s supposed to be a private matter. |
|  | there is no place for common room to play cards, to talk, to do a puzzle. And so, you're stuck making friends with your neighbours and going into their space, which I'm not really into that, that means I have to clean my place every damn time, hey, so I'm not into it. |
|  | had stairs and I didn't like the stairs. Being 120 lbs overweight it's really hard for me to take the stairs all the time |
|  | … my scooter. They can’t bring it upstairs, there is no elevator here, right? |
|  | Yeah, you’ve got elevators for people who have diseases. I'm in a disability unit, so I have a walk-in shower, there’s bars everywhere for me to grab onto. My – like I don't have anything below my waist that I have to worry about bending over to access. So it’s all, like, wheelchair accessible and – yeah. |
|  | There is (sic) only stairs, and I mean for anyone going up and down the stairs, if they’re elderly or young, they could accidentally mess up their knee. And I’ve had issues with my knees going up and down those stairs. |
|  | They have a wheelchair ramp and – there’s no elevator, mind you, but they have the first floor designated for people that have mobility issues. So they have all that in effect. Yes, there’s a safety ramp. |
|  | Yes, like I’m glad they put me on the second floor because I have back issues myself and pretty soon – like [I will need an] elevator…there’s going to be a lot of us that have, you know, issues like that that they can’t put us all on the first floor if there’s only 10 units on the first floor… they need to make entrances more accessible. In the front door, the ramp is up to the side, and the back ramp is not very good. It’s hard to – there’s a door that opens to the stairs, and it blocks the stairs. |
|  | Well, there are only two wheelchair places, so…out of 28 places there are only two that are wheelchair accessible, so they could easily have designed a few more of those. Although I don't know how a person, there’s so many people with scooters nowadays and also walkers. And so, none of these places are adaptable to, if I need mobility, I have to move down the hall or out, there’s no transition here… And so, these things aren’t flexible, they’re just made bingaty-bang, bangaty-boom, here you go. |
|  | R: Oh, my pet died, thank God.  I: Oh, I’m so sorry.  R: And other people in the building that had pets had to give them up.  I: Oh, that’s so sad.  R: And it broke (my) heart... It’s very heartless. |
|  | R: I think subsidized housing should allow pets. I'm going to put that on the record.  I: That you feel they should allow pets.  R: Yes, I miss my cats...and pets are very therapeutic, right?  I: Yes, I completely agree. |
|  | Sobriety, and just my mental health, right. I get anxiety being around most people, and you know, people are rude and disrespectful, and that affects your mental health, right, because people will say sh** or they’ll poke – you know... I realize I feel so much better and I feel more at peace when it’s just me and my cat. |
|  | ...The one woman [in my building] did have a pet and had to give it up because she was in a wheelchair and moved into the building. And for those people, my heart goes out to them because that’s what kept them alive. And now there’s nothing to keep them alive. |
| Reasonable housing costs | …because it’s getting so expensive with groceries and the rent... they’re raising [funding by] $125 in August which isn’t enough, but good enough to offset the price in place. |
|  | It’s just that those other things are costing more than, and you can’t get your rent much lower, I don’t think |
|  | the least rent I ever paid, ever |
|  | I never really have enough for food at the grocery store to buy there. And it seems like I’m running low on food. So that’s why I’m reaching out to get a food hamper almost every month, because it’s the option of getting food easily... I just have trouble saving money for food and going to the grocery store. I have rent to pay, and a phone bill and minutes... |
|  | I: OK. And does that feel like an amount that’s affordable for balancing your other living expenses? Your food and your other bills?  R: It’s never enough. |
| Mixed perceptions of physical and psychological safety | Well, it’s not necessarily like the building itself, it’s just the part of town that we’re in. But that’s (city) right now. Like, I had to call the RCMP [Royal Canadian Mounted Police] on a man outside yesterday who was smoking crack in the parking lot sort of thing, right, and like lingering around the building. Well, I was worried he was going to overdose too. |
|  | I wouldn’t want to be, like, outside late at night here very much kind of thing. But, like, the building itself, yes, but, like, psychological is a bit of a different story, I think. |
|  | It’s a mixed bag, right? I have kids that I’m actively involved with or whatever and so sobriety is obviously something that I’m struggling to do and aiming for…I’m out of the downtown core but there’s still a lot of drugs circulating the building. Even people like dealers...So it’s just hard when people knock on your door in the night, want you to score for them, or even during the day…And so I really had to sort of barricade my door in just between work and here and my kids, my decision, is really – I don’t even answer my knocks half the time because it’s just probably something that I don’t – that isn’t healthy to interact with. |
|  | …On the third floor they can’t hear what’s going on so it’s not always safe up there. And even on my floor, on the second floor, like I’ve had to call them many times saying, ‘There’s going to be a fight breaking out, you better get up here,’ yes or, ‘I’m concerned for this person’. |
|  | Like the one girl came out of her room and she’s standing there with like six knives in her hand not knowing where she is or what’s going on. And so we had to call the staff up there, and it’s like yes, she’s just clear out of her mind. She wasn’t aggressive or anything but she’s just really schizophrenic... |
|  | Ray: If I had known more about it, then maybe I’d reconsider.  Interviewer: Okay, why’s that?  Ray: It’s basically an addict’s paradise, if you know what I mean?  Interviewer: Okay, so lots of substance use?  Ray: Yes. |
|  | Kyle: It’s like preparing you for jail or something, I don’t know.  Interviewer: Okay, why do you say that?  Kyle: Doors slamming and – all the time and –  Interviewer: It’s noisy?  Kyle: Noisy, yes, gates, and just the rules and... |
|  | Oh, the way the building is built. It echoes a lot through the building. So anytime people are in the hallway, neighbours will obviously hear what they’re talking about and what they’re discussing, necessarily, even if it’s supposed to be a private matter. |
| The rules: Some stupid, some reasonable | doors slamming and – all the time…Noisy, yes, gates, and just the rules… |
|  | I: What kind of rules are there?  R: In our building, there's no men or significant others allowed. The children have to be monitored at all times and your key is your key. You're not allowed to give it to anybody else. The security at this building is amazing.  I: And so, do you find these rules helpful to you?  R: Absolutely. |
|  | guest rules are kind of like the annoying, like, most annoying rules. |
|  | Yes, there is rules…like, our visiting hours are from 10:00 a.m. to 7:00 p.m. I can’t have – like, I have a daughter and grandchildren so if they came to town, I wouldn't be able to have them overnight or nothing like that. And that's not – like, they don't have those types of rules over at the other place I was trying to get into. And that's my – basically, other than that, that's the only thing I don't really care for. |
|  | I don’t remember the time when it started…but this is what I was told by people that have been here longer. But it went to 11 p.m. at night that the guests had to be kicked out. Whereas right now it’s 10 a.m. to 7 p.m. And like that’s frustrating because 7 p.m. does – like, it kind of sucks because I'm not a morning person. I think a lot of other people that are here aren’t really morning people, per se. |
|  | I was informed by one of the workers from (non-profit organization) as well as another business in town that deals with addiction and people who have severe mental health. So I understand at the beginning there we signed our tenancy agreement about the visitors, it being COVID and all that that we wouldn’t have visitors right away considering COVID. But then as soon as that died down we would be able to have our – visitors would be there. Well, so no, we’re still not allowed visitors in our units at all and I’m not quite sure why. |
|  | you have to stay with your guests the entire time they’re here. So, you have to walk them to and from the door. No problem with that…You have to attend a weekly, like, floor meeting. No problem with that either |
|  | Once every three months, we do an inspection and every day we have to check in with that, or else they come check on us. |
|  | It’s pretty lax, and it’s basically just the usual stuff, be respectful towards people, or you could get evicted kind of thing. Your substance use can’t affect others in the building, that sort of thing. So, no, I think they’re all…very reasonable rules. |
|  | [housing administers] can change the rules, our tenancy agreement, they can do it whenever they want. They can change any aspect of that without notifying us and anything is subject to change without notice |
|  | Basically, they can kick us out whenever they feel like it and actually take our key cards away and say, “Okay, you’re kicked out for two days, then you can come back.’ They will let you back in. |
|  | no fires on the patios and stuff like that, just typical, no laundry flapping and no dogs, no barking, and so it makes it a really quiet environment |
|  | Rules and guidelines are helpful because they maintain a good, healthy, living environment. |
|  | [the rules were] very strict, extremely strict. Like for a person – I have managed for 30 years apartment blocks, and to go into a situation like the one I’m in it’s hideous. It’s totally hideous and unacceptable. |
|  | We’re all alone, single tenants, and not one – we’re not allowed to have company like stay overs… You can’t hang pictures on the wall, you can’t use nails. If you use a thumbtack and it makes too big a hole you could charged for it. It’s just hideous. It’s just hideous. |
|  | [the building rules are] good for the most part, (although) they do have double standards here. There’s favouritism. Not everybody gets the same rules, it seems. But we all deal with it. |
|  | R: Certain rules, yes. Yes, I can see the necessity of some of them but like with any...I think it might be privately owned, so there’s usually some weird bias rules that come up that don’t last long because they’re pretty – what’s the word? Stupid.  I: So it sounds like maybe the rules change pretty frequently.  R: Yes. |
| Perceptions of compliance and punishment | You know, there’s favouritism. And there’s compliance, non-compliance. And punishment. Yeah. I've been on two citations already. Right. And I found it unnecessary actually. Because it had nothing to do with me being violent or anything, right, or having me cause any mayhem. I just think management are trying to make decisions. The ACT team. The ACT team is here for medications. They’re like the medication cops. They...make sure you don’t get bad handouts. |
|  | need to try to refrain from drugs and alcohol |
|  | No males here. And no drinking, no partying and that kind of thing, you know. |
|  | And then, you know, you do your one-on-one meetings every week with a worker. And you do your residential floor meetings every week. And those are the only two mandatories in order to stay here. |
|  | The biggest one is not to have any males on the property over the age of 18…And that’s an immediate eviction |
|  | I think there’s people in here that should be not necessarily kicked out, but even then, considered to be kicked out... even though there is no additional bias and there’s fairness, it’s still some people get a lesser degree of severity. But there’s people here that should be on clear disciplinary effects, right? Like... if you’re really bad, they kick you out for a number of days and stuff like that... But I’ve witnessed things where the person should have in like – you know, it’s been repeat, or there’s been interactions I’ve witnessed, and the person should have at least...be under that kind of consideration. Because they haven’t received any kind of – and it’s just like abuse to the staff and all sorts of stuff like that. |
| Internalized readiness requirements and housing disparate groups in a market marked by scarcity | There needs to be more (supportive) in (city), yes, because our city is growing. There’s a lot more younger people and people that are just graduating and going to move out – like jobs that pay a good wage, don’t pay for rent and food and bills. For an average place in (city) right now it’s like $1,900 for a studio and we need better, cheaper and easier accessible affordable housing in (city), which is a big thing. |
|  | They need to make a lot more buildings like this...We need a whole bunch more. |
|  | I really think with, like, second stage supportive housing there needs to be a lot more for women in this province. We're lucky to have another one getting built at (city), but when I was looking, like, province-wide there’s hardly anything. I also feel very strongly that there needs to be more housing for people in active addiction, with harm reduction being a short-term goal and recovery being a long-term goal. |
|  | They need to have more second stage programs after treatment or even without treatment or second stage. And like [the] halfway house thing where you live in a house, then you have to be actively seeking employment, you have to stay sober, and stuff like that. |
|  | We need more second-stage housing. Like housing that – and even third stage, whatever that looks like. Basically, a whole remapping or revamping of the housing system where they actually have places like more supports like this for people who are trying to stay clean, who are sober, or trying to remain sober, or trying to get sober. |
|  | now so there’s two (facilities) – there’s the (building name) where it’s supposed to the stepping stone in order to get into my housing, into (building name) where I live. |
|  | And they’re actually putting up a second one (building), which is too bad because we need one for men too. Not necessarily fleeing abuse, although it does happen, but just men with kids that are in housing parameters that they can’t fill, they need to have one, because they already have (building) and they’re building a second one |
|  | get more housing for seniors |
|  | Why isn’t there supportive housing for two people? |
|  | Well, you know what, it kind of depends on their needs actually because I have had some people with, like, children – like, people have different experiences here based on what they need, right? Like for me, a single person, it's totally awesome, but if you have like – for parents with kids and stuff like that, it might not be totally the best just because they don't always get the space that they need. So, I would recommend it if you, like, are desperate though, right? Of course. |
